# Beam emittance preservation using Gaussian density ramps in a beam-driven plasma wakefield accelerator

**DOI:** 10.1098/rsta.2018.0181

**Published:** 2019-06-24

**Authors:** M. D. Litos, R. Ariniello, C. E. Doss, K. Hunt-Stone, J. R. Cary

**Affiliations:** 1 Center for Integrated Plasma Studies, Department of Physics, University of Colorado Boulder, Boulder, CO, USA; 2 Tech-X, Boulder, CO, USA

**Keywords:** plasma wakefield accelerator beam, emittance preservation matching, Gaussian density ramp

## Abstract

A current challenge that is facing the plasma wakefield accelerator (PWFA) community is transverse beam emittance preservation. This can be achieved by balancing the natural divergence of the beam against the strong focusing force provided by the PWFA plasma source in a scheme referred to as beam matching. One method to accomplish beam matching is through the gradual focusing of a beam with a plasma density ramp leading into the bulk plasma. Here, the beam dynamics in a Gaussian plasma density ramp are considered, and an empirical formula is identified that gives the ramp length and beam vacuum waist location needed to achieve near-perfect matching. The method uses only the beam vacuum waist beta function as an input. Numerical studies show that the Gaussian ramp focusing formula is robust for beta function demagnification factors spanning more than an order of magnitude with experimentally favourable tolerances for future PWFA research facilities.

This article is part of the Theo Murphy meeting issue ‘Directions in particle beam-driven plasma wakefield acceleration’.

The luminosity demanded of a future high-energy lepton collider will require the delivery of beams with nm-rad normalized emittance and energies upwards of 1 TeV [1,2]. The plasma wakefield accelerator (PWFA) is an advanced particle accelerator that has the potential to meet these demands at a reduced size and cost compared with metallic structure-based accelerators [3,4]. To date, multi-GeV energy gain in a single-stage beam-driven PWFA [5] has been repeatedly achieved in experiment for both electron and positron beams [6–9]. Beam emittance preservation, however, has yet to be demonstrated by the plasma accelerator community. Here, a simple empirical formula is presented that describes the precise beam and plasma conditions required to provide near-perfect matching of a beam into a PWFA stage using a Gaussian plasma density ramp. In addition, numerical studies are carried out to show that the experimental tolerances are well within the reach of next-generation research facilities.

This study considers a PWFA operating in the nonlinear blowout regime [10], wherein a plasma wake is driven by a dense, ultrarelativistic electron beam. The plasma wake-driving beam is referred to as the drive beam and the beam that is accelerated in the plasma wake (also ultrarelativistic) is referred to as the witness beam. The witness beam must be matched to the plasma of a PWFA in order to preserve its rms normalized emittance *ε*_*n*_≡*γ*_*b*_ *ε*, where *γ*_*b*_ is the relativistic Lorentz factor of the beam, and $\varepsilon \equiv \sqrt{\langle x^{2}\rangle \langle x^{\prime 2}\rangle -{\left\langle xx^{\prime }\right\rangle }^{2}}$ is the root-mean-square (rms) geometric emittance [11]. *x* represents the horizontal position of an individual particle in the beam and *x*′≡*p*_*x*_/*p*_*z*_ is the angle of the trajectory of an individual particle. *p*_*x*_ and *p*_*z*_ are the particle's horizontal and longitudinal components of momentum, respectively. A beam is considered matched to the plasma when its transverse envelope avoids the characteristic beating associated with betatron oscillations [12,13]. The transverse envelope is characterized by the rms spot size along each transverse dimension, defined as $\sigma_{x}\equiv \sqrt{\langle x^{2}\rangle -{\left\langle x\right\rangle }^{2}}$ in the *x*-plane, and similarly for the *y*-plane. Matching occurs when the linear focusing force provided by the ion column in a blowout plasma wake is balanced against the divergence of the beam.

A mismatched beam with a finite energy spread undergoes chromatic filamentation inside a PWFA, leading to emittance growth and transverse profile distortion. Fortunately, filamentation can be avoided by focusing the beam to the properly matched size at the start of the PWFA plasma source. Typical electron beam sizes in a conventional accelerator are much larger than the matched size required of a PWFA, however. There have been studies that aim to design conventional electromagnetic beam delivery systems that can effectively transport a beam from one plasma stage to the next [14–16], but it remains a significant challenge to provide the necessary focusing within a reasonable distance. To address this challenge, the strong focusing forces arising inside the plasma wake itself can be used in a plasma density ramp in order to match the beam into the plasma accelerator [17–24]. The same technique can be employed to control the divergence at the exit of the plasma source in order to prevent normalized emittance growth during free diffraction [25,26].

## 1. Beam matching

An electron beam is matched to a uniform-density plasma if the beam envelope experiences no oscillations, requiring that *α* =  − (1/2)(d*β*/d*z*) = 0, which occurs when *β* = *β*_*m*_ = *k*^−1^_*β*_, *α* = *α*_*m*_ = 0 and *γ* = *γ*_*m*_ = *k*_*β*_, where *k*_*β*_ is the betatron wave number. Here, *β*, *α* and *γ* represent the Courant–Snyder parameters [27] of the beam, while *β*_*m*_, *α*_*m*_ and *γ*_*m*_ represent the parameters that satisfy the matching condition inside the plasma. The matched rms transverse size of the beam is then given by $\sigma_{m}=\sqrt{\varepsilon \beta_{m}}=\sqrt{\varepsilon /k_{\beta }}$. The betatron radial frequency is $\omega_{\beta }=\omega_{p}/\sqrt{2\gamma_{b}}=c\;k_{\beta }$, where *ω*_*p*_ = (*n*_*p*_*e*^2^/*ϵ*_0_*m*_*e*_)^1/2^ is the plasma electron angular frequency, *n*_*p*_ is the plasma electron number density, *ϵ*_0_ is the vacuum permittivity, *m*_*e*_ is the electron mass, *e* is the elementary charge and *c* is the speed of light in a vacuum.

Small longitudinal variations of the plasma density *n*_*p*_ and/or the beam Lorentz factor *γ*_*b*_ lead to the approximation *k*_*β*_≃*k*_*β*,0_(1 − *δ*/2), where *δ*≡*δ*_*n*_ − *δ*_*γ*_ − (3/4)*δ*^2^_*n*_ + (9/4)*δ*^2^_*γ*_ − (1/2)*δ*_*n*_*δ*_*γ*_ to second order in *δ*_*n*_ and *δ*_*γ*_. Here *δ*_*n*_≡Δ*n*_*p*_/*n*_*p*,0_≪1, *δ*_*γ*_≡Δ*γ*_*b*_/*γ*_*b*,0_≪1, *n*_*p*,0_ and *γ*_*b*,0_ are the nominal plasma density and beam momentum, and *k*_*β*,0_ is the nominal value of *k*_*β*_ when *δ* = 0. The relative emittance growth over a distance ∼*k*^−1^_*β*,0_ for an initially matched beam traversing a plasma with a density perturbation is then *δ*_*ε*_≡(Δ*ε*(*k*^−1^_*β*_)/*ε*_0_)≃(*δ*^2^_*n*_ + *σ*^2^_*δ*_*γ*__), to second order in *δ*_*n*_ and *σ*_*δ*_*γ*__, where *σ*_*δ*_*γ*__ = (〈*δ*^2^_*γ*_〉)^1/2^ is the rms relative energy spread of the beam, and where *ε*_0_ is the initial matched emittance. It is evident that beam emittance can be well preserved under an adiabatic transition of the plasma density, providing a method for beam matching into or out of a PWFA plasma source [17,20,21]. The adiabatic condition is (1/2)|(d*β*_*m*_/d*z*)| = (4*n*_*p*_*k*_*β*_)^−1^|(d*n*_*p*_/d*z*)|≪1 [22,24], where *γ*_*b*,0_ is assumed to be a constant of the motion. Under this assumption, the adiabatic condition depends only on the plasma density profile and not on the beam evolution.

Beam matching into a uniform plasma density may also be achieved with plasma density gradients that exceed the adiabatic limit, even though the beam may not be matched to the local plasma density throughout the ramp. This can be leveraged to reduce the overall scale of the beam-matching section of plasma. Similar schemes that use a highly mismatched beam with an adiabatic ramp can also reduce the length scale of the matching section [28,29]. This study concentrates on matching into a uniform plasma with a Gaussian plasma density ramp shape, which is not fully adiabatic. It is chosen because it provides a continuous density profile at the transition to the bulk plasma region and it rapidly reduces to negligible densities away from the bulk. It has also been used to model the heat-pipe oven plasma sources used in PWFA experiments at SLAC National Accelerator Laboratory's Facility for Advanced Accelerator Experimental Tests (FACET) [18]. The Gaussian plasma density ramp takes the form *n*_*p*_(*z*) = *n*_*p*,0_ *e*^−(*z*^2^/2*σ*^2^) ^, where the endpoint of the uniform density region of the bulk plasma is located at *z* = 0, and the half-width of the ramp is $\sigma_{\mathrm{hw}}=\sqrt{2\ln2}\;\sigma$.

**Figure 1.**
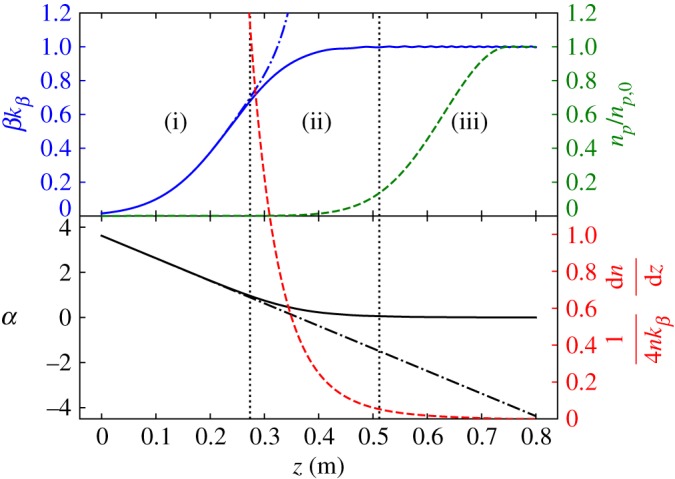
Evolution of a beam in a Gaussian plasma density ramp along *z*. Three regions of evolution are separated by the vertical dotted black lines and are identified: (i) vacuum-like, (ii) non-adiabatic and (iii) adiabatic. Top: betatron evolution of the beam and plasma density profile. Solid blue line: evolution of *β*(*z*)*k*_*β*_(*z*) in the plasma. Dot-dashed blue line: vacuum-like evolution of *β*(*z*)*k*_*β*_(*z*). Solid green line: plasma density *n*_*p*_(*z*). Bottom: evolution of alpha parameter and adiabaticity parameter. Solid black line: evolution of *α*(*z*) in plasma. Dot-dashed black line: vacuum-like evolution of *α*(*z*). Dashed red line: adiabaticity parameter (4*n*_*p*_(*z*)*k*_*β*_(*z*))^−1^(d*n*_*p*_(*z*)d*z*).

The Gaussian plasma ramp satisfies the adiabatic condition for |*z*|≲2*σ*. In this region, the beam will remain matched to the local plasma density if it is initially matched. In the region 2*σ*≲|*z*|≲4*σ*, the ramp is non-adiabatic, and the beam is not matched to the local plasma density. In the region 4*σ*≲|*z*|, the local matched betatron wavelength is much larger than the beam's beta function, *βk*_*β*_≪1, and the beam evolution can be approximated as being vacuum-like, due to the small, slow effect of the plasma on the beam. Figure 1 shows the evolution of a beam that is matched to a uniform density plasma by a Gaussian ramp, and identifies the three qualitatively different regions of beam evolution.

A mismatched beam will experience chromatic emittance growth in a plasma via filamentation, reaching a saturated limit after many betatron periods. In a uniform density, the parameter *B*_*m*_≡(1/2)(*β*_*i*_*γ*_*m*_ − 2*α*_*i*_*α*_*m*_ + *γ*_*i*_*β*_*m*_) gives the ratio of the saturated normalized emittance of a fully filamented beam to the beam's initial normalized emittance [19,28–32]. Here, *β*_*i*_, *α*_*i*_ and *γ*_*i*_ represent the initial Courant–Snyder parameters of the beam. In addition to the rms emittance growth, the distribution of the beam particles in transverse phase space will become distorted, causing halo formation around a strongly peaked, non-Gaussian core.

A beam that is bi-Gaussian in transverse phase space (*x*, *x*′) has a density function *f*(*J*) = *ε*^−1^*e*^−*J*/*ε*^, where *J* is the betatron action amplitude. The phase space coordinates (*x*, *x*′) are related to the generalized action-angle coordinates (*J*, *ϕ*) by the transformations $x=\sqrt{2J\beta }\cos\phi$ and $x^{\prime }=-\sqrt{2J/\beta }(\sin\phi +\alpha \cos\phi )$. The kurtosis of *J* for a bi-Gaussian beam is *κ*(*J*) = 9. The process of filamentation in a plasma acts to increase *κ*(*J*) [30]. For nearly matched beams (*B*_*m*_≃1), the saturated kurtosis is given by *κ*(*J*)≃*B*_*m*_ 9. For significantly mismatched beams (*B*_*m*_≫1), the kurtosis approaches an asymptotic upper limit of 15. When the beam is at a waist, the kurtosis of *J* will be most evident in the *x*′ projection of the beam; when it is far from a waist, it will be most evident in the *x* projection of the beam. The effect is to create a sharp peak at the centre of the distribution, with wide, halo-like tails extending well beyond the rms spot size.

## 2. Numerical beam-matching studies

The evolution of the beam's beta function as it travels through a plasma in a nonlinear blowout wake is described by the equation

2.1 $$\frac{1}{2}\beta^{\prime \prime }(z)+k_{\beta }^{2}(z)\beta (z)-\frac{1}{\beta (z)}\left(1+{\left(\frac{\beta^{\prime }(z)}{2}\right)}^{2}\right)=0.$$

An analytical solution for *β*(*z*) cannot generally be found for an arbitrary plasma density profile. It is, therefore, necessary to use numerical methods to study the evolution of the beam through a given plasma density profile. This exercise bears some resemblance to the matching of a high intensity laser pulse into a laser wakefield accelerator plasma source [33].

**Figure 2.**
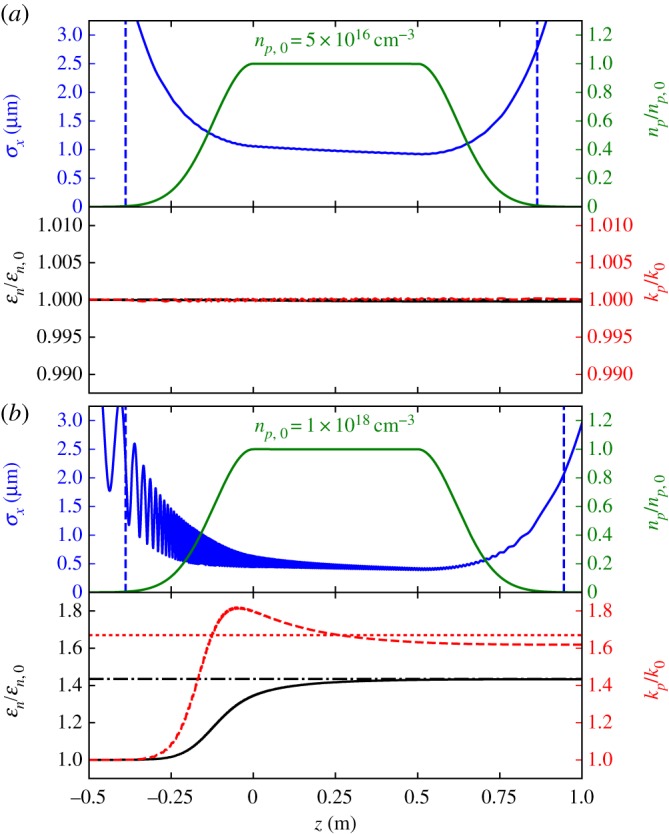
Evolution of a 10 GeV electron beam with initial normalized emittance of *ε*_*n*,0_ = 5 mm mrad through two different plasma sources. Both plasma sources have identical relative density profiles with bulk densities of (*a*) 5 × 10^16^ cm^−3^ and (*b*) 1 × 10^18^ cm^−3^. The beam propagates in the positive *z*-direction and is focused to achieve perfect matching to the bulk plasma in (*a*), and is under-focused in (*b*). The green line indicates the normalized plasma density profile *n*_*p*_/*n*_*p*,0_; the blue line indicates the rms beam spot size *σ*_*x*_; the dashed blue lines indicate the virtual vacuum waist location at the entrance and exit of the plasma; the solid black line indicates the relative growth of the normalized rms emittance of the beam *ε*_*n*_/*ε*_*n*,0_; and the dashed red line indicates the relative growth of the kurtosis of the beam's betatron action amplitude *κ*/*κ*_0_. Both the emittance and the kurtosis are scaled relative to their respective initial values. The black dot-dashed line indicates the value of *B*_*m*_ calculated at the start of the plasma bulk, and the dotted red line indicates the maximum possible growth for *κ*.

Figure 2 shows the numerically calculated evolution of a 10 GeV electron beam with an initial normalized emittance of *ε*_*n*_ = 5 mm mrad, a flat relative energy spread with a half-width of *σ*_*δ*_*p*_,hw_ = 0.01, and a beta function at the virtual vacuum waist of β*_v_ = 10 cm passing through two different plasma sources. The virtual vacuum waist here refers to the beam waist size and waist position that would be realized in the total absence of plasma. The virtual vacuum waist is numerically calculated from the initial and final beam parameters far away from the plasma source. The initial beam parameters are chosen to approximate those expected at the next-generation experimental facility FACET-II at the SLAC National Accelerator Laboratory [34,35].

A bi-Gaussian distribution of 10^6^ electrons is numerically propagated through each plasma source in figure 2 using a step size $\Delta z_{\mathrm{step}}=k_{p,0}^{-1}\sqrt{2\gamma_{b,i}}/20$, where *γ*_*b*,*i*_ is the initial relativistic Lorentz factor of the beam. The transverse motion of each electron is calculated using Hill's Equation: *x*′^′^ + *K*(*z*)*x* = 0, where *K*(*z*) = (*e*^2^/2*γ*_*b*_*m*_*e*_*c*^2^*ϵ*_0_) *n*_*p*_(*z*) arises from the linear focusing force produced by the pure ion column of density *n*_*p*_(*z*) inside the nonlinear plasma wake [36]. The centroid energy gain (or loss) rate is taken into account at each point in the calculation using a formula derived from basic physics arguments justified in the appendix:

2.2 $$\frac{d\gamma_{b}}{dz}=G_{0}\frac{k_{p}(z)}{k_{p,0}}\left(2\frac{k_{p}(z)}{k_{p,0}}-1\right),$$

where *G*_0_ is the constant rate of energy gain inside the bulk region of the PWFA, and *k*_*p*,0_ represents the nominal plasma wave number in the uniform density region of the plasma. The energy gain rate in the bulk region of the plasma is set to $G_{0}=(16.7\;\mathrm{GeV}\;m^{-1}/m_{e}c^{2})\sqrt{n_{p,0}/5\times 10^{16}\;{\mathrm{cm}}^{-3}}$, based on results obtained from the three-dimensional particle-in-cell (PIC) simulations described in the appendix. The energy spread is kept fixed in these calculations, as growth in energy spread is beyond the scope of this study. Regardless of the energy spread, the emittance growth is minimized when the centroid energy of the beam is matched to the plasma. For parameters relevant to this study, the total change in centroid energy over the full distance of the Gaussian plasma density ramp is typically less than 10% of the initial beam energy, which is inconsequential for matching purposes.

Figure 2*a* shows near-perfect matching of the beam into the bulk plasma source, where the normalized beam emittance *ε*_*n*_ and kurtosis of the betatron action amplitude *κ*(*J*) grow by significantly less than one per cent, agreeing with the prediction *B*_*m*_ = 1.00. In figure 2*b*, the beam is under-focused upon entering the bulk plasma, resulting in filamentation that reaches saturation shortly after entering the bulk. In this case, the emittance grows by 43.2% compared with the initial value, agreeing with the *B*_*m*_ = 1.43 prediction. The kurtosis also grows significantly, nearly reaching the asymptotic maximum limit, even while *B*_*m*_ is less than 2.

**Figure 3.**
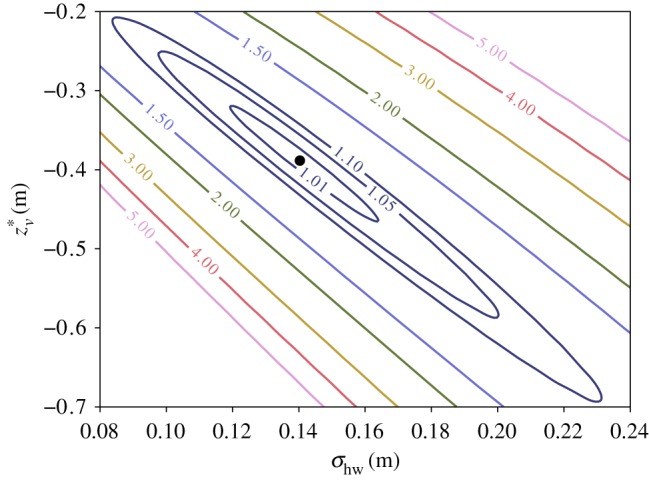
Contour map of *B*_*m*_ calculated at the start of the uniform density bulk region of the plasma as a function of the virtual vacuum waist location z*_v_ and the Gaussian plasma density ramp half-width *σ*_hw_ for the same beam used in figure 2 and a plasma bulk density of *n*_*p*,0_ = 5 × 10^16^ cm^−3^. The exact matching condition where *B*_*m*_ = 1 is indicated by the black dot.

It is useful to identify the experimental conditions that will yield beam matching for a given input beam and bulk plasma density. It is also important to understand the sensitivity of the emittance growth to the experimental parameters involved. To perform these tasks, *B*_*m*_ is calculated over a range of values for the entrance ramp half-width *σ*_hw_ and the virtual vacuum waist location z*_v_. The beam beta function is numerically propagated through the plasma ramp to the start of the bulk, where *B*_*m*_ is then calculated. Figure 3 shows a contour map of *B*_*m*_ obtained for the same 10 GeV electron beam used in figure 2 entering a plasma source with a bulk density of *n*_*p*,0_ = 5 × 10^16^ cm^−3^. The matching point is found to be (*σ*_hw, *m*_, z*_v,*m*_) = (14.0 cm,  − 38.7 cm) and is indicated on the figure with a black dot.

The *B*_*m*_ = 1.01 contour in figure 3 signifies 1% emittance and kurtosis growth at saturation and spans a 4.45 cm range of ramp half-widths, each with a corresponding optimal z*_v_. In practice, the virtual vacuum waist location will be determined by an electromagnetic beam delivery system with centimetre-scale precision [34]. Therefore, the beam emittance can be preserved by tuning the vacuum waist location to accommodate for error in the plasma ramp width. If β*_v_ = 10 cm, then z*_v_ needs to change by 3.24 cm for every 1 cm that *σ*_hw_ deviates from the optimal matching value.

## 3. Empirical matching formula

By expanding the numerical study to explore a wide range of beam energy *γ*_*b*,0_ and bulk plasma density *n*_*p*,0_, an empirical matching formula emerges that depends only on the vacuum waist beta function β*_v_. The scalings for the optimal Gaussian ramp half-width and virtual vacuum waist location are given by quadratic equations:

3.1 $$\begin{array}{rl} {\tilde{\sigma }}_{\mathrm{hw}} & =1.96\times 10^{-3}\;{\tilde{\beta }}_{v}^{*2}+1.49\;{\tilde{\beta }}_{v}^{*}-3.08\\ \;\mathrm{and}\;\;{\tilde{z}}_{v}^{*} & =-1.47\times 10^{-2}{\tilde{\beta }}_{v}^{*2}-4.34\;{\tilde{\beta }}_{v}^{*}+17.9. \end{array}\}$$

All variables are normalized by a factor $k_{\beta ,0}=(1.33\times 10^{-4}\;m^{-1})\sqrt{n_{p,0}\;[cm^{-3}]/\gamma_{b,0}}$ (indicated by tildes). Here, *n*_*p*,0_ is the bulk plasma density, and *γ*_*b*,0_ is the Lorentz factor of the beam at the start of the bulk plasma. Because the energy gain that occurs in the ramp is small, it is possible to approximate *γ*_*b*,0_ as constant without significantly impacting the matching quality.

**Figure 4.**
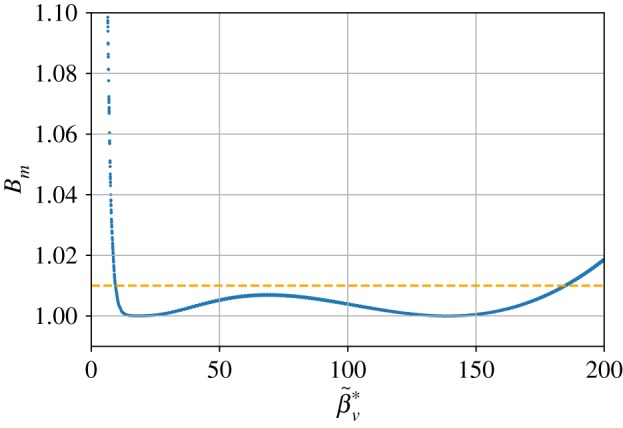
Calculated value of *B*_*m*_ using parameters obtained from the ramp and waist optimization formula given in equation (3.1) for a range of ${\tilde{\beta }}_{v}^{*}$.

The scope of validity for this formula was found by calculating the degree of matching, *B*_*m*_, over a wide range of the normalized virtual vacuum waist beta function, ${\tilde{\beta }}_{v}^{*}$. The scan covered a span of 201 × 201 evenly spaced working points in the parameters space defined by 5 cm ≤ β*_v_ ≤ 85 cm and 2.8 × 10^15^ cm^−3^ ≤ *n*_*p*,0_ ≤ 3.6 × 10^18^ cm^−3^, and a 10 GeV beam was assumed. The ramp half-width and vacuum waist location used in each iteration were determined according to equation (3.1) for every computational working point. The value of ${\tilde{\beta }}_{v}^{*}$ was calculated for each point, as was the value of *B*_*m*_ at the peak density location (i.e. end of the ramp). Figure 4 shows the resultant value of *B*_*m*_ that is achieved by the formula presented in equation (3.1) over the considered range of ${\tilde{\beta }}_{v}^{*}$. The formula preserves emittance to a degree of 1% (*B*_*m*_ ≤ 1.01) over a range of ${\tilde{\beta }}_{v}^{*}$ from 9.50 to 185. When the emittance is preserved, the beam's vacuum waist spot size is demagnified by a factor $\sqrt{{\tilde{\beta }}_{v}^{*}}$ at the end of the plasma entrance ramp. Therefore, the empirical matching formula presented here is valid for a range of spot size demagnification from 3.08 to 13.6. The same optimization scalings can be used to tailor the dynamics of the beam as it exits the plasma source, given the desired value of the virtual vacuum waist ${\tilde{\beta }}_{v}^{*}$ at the plasma exit.

## 4. Conclusion

Beam emittance and profile quality can be preserved in a PWFA through the use of multi-centimetre-long Gaussian density ramps. Numerical studies reveal an empirical quadratic formula to predict the optimal ramp length and vacuum waist position for matching that depends only on the vacuum waist beta function. The empirical matching formula is shown to work over a wide range of conditions with experimentally favourable tolerances. This, along with the compact and passive nature of the Gaussian plasma density ramps, makes them an attractive solution for beam matching and emittance preservation in PWFA experiments and applications.

## Data accessibility

This article has no additional data.

## Appendix A

The longitudinal phase of the witness beam within the wake changes continuously while traversing the ramps. The beam is in the accelerating phase at higher density, and in the decelerating phase at lower density. Equation (2.2) shows that energy gain will occur within a distance of $\sqrt{2}\;\sigma_{\mathrm{hw}}$ from the bulk plasma in a Gaussian plasma density ramp. Beyond this distance, the witness beam is losing energy, though the rapid decrease of the plasma density mitigates the total energy loss. The net energy gain is 0.62 *σ*_hw_ *G*_0_ in a single Gaussian density ramp.

The form of equation (2.2) results from the following assumptions: (i) the witness beam remains a fixed distance behind the drive beam, (ii) the longitudinal wakefield has a linear slope along the dimension *ct* − *z*, (iii) the slope of the wakefield and the length of the plasma wave scale linearly with *k*_*p*_ and (iv) the boundary conditions (d*γ*_*b*_/d*z*) = *G*_0_ in the uniform density bulk plasma and (d*γ*_*b*_/d*z*) = 0 where the plasma density reaches zero are satisfied. The energy gain model agrees (rms error 0.06 *G*_0_) with the accelerating gradient measured in a number of short, three-dimensional simulations carried out over two orders of magnitude of plasma density using a PIC code.

The PIC code VSim [37] was used to simulate a PWFA modelled after the design parameters for FACET-II [34,35]. A total of 13 moving window three-dimensional simulations of 425 μm in length were performed at different plasma densities. The sampled plasma densities ranged from 5 × 10^14^ cm^−3^ to 5 × 10^16^ cm^−3^ in approximately logarithmically spaced increments. The parameters for the drive and witness beam are listed in table 1. The rms transverse size of each bunch is set to the matched size for a given plasma density, which agrees within 1% of the value predicted by our numerical beam matching model. Simulation parameters are listed in table 2.

**Table 1.** Beam parameters used in PIC simulations.

parameter	drive beam	witness beam
initial energy	10 GeV	10 GeV
bunch charge	1.5 nC	0.5 nC
RMS bunch length	5.2 μm	18 μm
normalized emittance	5.3 mm-mrad	7.0 mm-mrad
inter-bunch spacing	135 μm

**Table 2.** Three-dimensional PIC simulation parameters.

parameter	*x*, *y*	*z*
theadend simulation size	250–1250 μm	250 μm
cell size	0.9–4.6 μm	1.0 μm
number of cells	274	250
plasma macro particles per cell	1	
beam macro particles per cell	8	
step length	2.04 × 10^−15^ s	

The value of the loaded longitudinal wakefield is extracted from each simulation and compared with the energy modulation model in equation (2.1). The rms error of the instantaneous rate of energy gain *G*(*z*) = *eE*(*z*) is calculated to be 0.06 *G*_0_, where *E*(*z*) is the instantaneous longitudinal wakefield acting on the witness beam, and *G*_0_ is the rate of energy gain for the witness beam at the highest sampled density, representing the bulk density in this study. The rms error is defined as ${\left(\sum {\left(E_{\mathrm{mod},i}-E_{\mathrm{sim},i}\right)}^{2}/N\right)}^{1/2}$, where *N* is the number of sampled plasma densities, and *E*_mod, *i*_ and *E*_sim, *i*_ are the longitudinal electric wakefield for plasma density *i* from the model and the simulation, respectively.
